# SP-B and SP-C Containing New Synthetic Surfactant for Treatment of Extremely Immature Lamb Lung

**DOI:** 10.1371/journal.pone.0039392

**Published:** 2012-07-13

**Authors:** Atsuyasu Sato, Machiko Ikegami

**Affiliations:** Division of Pulmonary Biology, Cincinnati Children's Hospital Medical Center, University of Cincinnati, Cincinnati, Ohio, United States of America; The Ohio State Unversity, United States of America

## Abstract

Although superiority of synthetic surfactant over animal-driven surfactant has been known, there is no synthetic surfactant commercially available at present. Many trials have been made to develop synthetic surfactant comparable in function to animal-driven surfactant. The efficacy of treatment with a new synthetic surfactant (CHF5633) containing dipalmitoylphosphatidylcholine, phosphatidylglycerol, SP-B analog, and SP-C analog was evaluated using immature newborn lamb model and compared with animal lung tissue-based surfactant Survanta. Lambs were treated with a clinical dose of 200 mg/kg CHF5633, 100 mg/kg Survanta, or air after 15 min initial ventilation. All the lambs treated with air died of respiratory distress within 90 min of age. During a 5 h study period, Pco_2_ was maintained at 55 mmHg with 24 cmH_2_O peak inspiratory pressure for both groups. The preterm newborn lamb lung functions were dramatically improved by CHF5633 treatment. Slight, but significant superiority of CHF5633 over Survanta was demonstrated in tidal volume at 20 min and dynamic lung compliance at 20 and 300 min. The ultrastructure of CHF5633 was large with uniquely aggregated lipid particles. Increased uptake of CHF5633 by alveolar monocytes for catabolism was demonstrated by microphotograph, which might be associated with the higher treatment dose of CHF5633. The higher catabolism of CHF5633 was also suggested by the similar amount of surfactant lipid in bronchoalveolar lavage fluid (BALF) between CHF5633 and Survanta groups, despite the 2-fold higher treatment dose of CHF5633. Under the present ventilation protocol, lung inflammation was minimal for both groups, evaluated by inflammatory cell numbers in BALF and expression of IL-1β, IL-6, IL-8, and TNFα mRNA in the lung tissue. In conclusion, the new synthetic surfactant CHF5633 was effective in treating extremely immature newborn lambs with surfactant deficiency during the 5 h study period.

## Introduction

The current commercial surfactants for treatment of preterm newborns with respiratory distress syndrome (RDS) are derived from lung tissue or bronchoalveolar lavage fluid (BALF) of bovine or swine. Clinical responses to surfactant treatment reveal the superiority of animal-derived surfactant over synthetic surfactant [1], including Surfaxin and Exosurf, whereas some weak points of animal-derived surfactant have been indicated, including limited supply, posing a danger of infection, and inevitable batch-to-batch differences in function. Moreover, use of synthetic surfactant would lower the cost for treatment. For over 2 decades, many trials have been made to develop synthetic surfactant comparable in function to animal-driven surfactant. CHF5633 is the new totally synthetic surfactant synthesized by Chiesi Farmaceutici S.p.A (Parma, Italy) for the treatment of preterm newborn infants with RDS. CHF5633 contains R-dipalmitoylphosphatidylcholine (DPPC) and 1-palmitoyl-2oleoyl-glycero-3-phospho-1-glycerol (POPG) 1∶1 ratio (98.3%), surfactant protein (SP)-C analogue (1.5%), and SP-B analogue (0.2%). SP-C analogue is a 33-amino acid protein formed by both N-terminal segment analog of native SP-C and hydrophobic C-terminal helical segment similar to that of native SP-C. Amino acid sequence of SP-C analog is IPSSPVHLKRLKLLLLLLLLILLLILGALLLGL. SP-B analogue is a 34-amino acid protein derived from the two parts (8–25 and 63–78) of the full-length natural SP-B. Preliminary unpublished studies with CHF5633 demonstrated high surface activity as well as resistance against surfactant deterioration in vitro and improvement of preterm newborn rabbit lung compliance. CHF5633 is a white uniform suspension, stable at room temperature, has low viscosity, and does not require any heating before use.

The lung injury occurs when extremely preterm newborns are resuscitated by manual ventilation in the delivery room. After preterm newborns are transferred to the neonatal intensive care unit, surfactant treatment is given as early as possible to minimize lung injury caused by resuscitation [2], improve surfactant distribution [3], and minimize inhibition of surfactant function by leaked proteins [4], [5], [6]. In clinical practice, surfactant treatment is typically administered within 20 min after birth.

The object of this study was to evaluate the efficacy of the treatment with CHF5633 in comparison with Survanta® (Abbott Nutrition, Columbus, OH) using the preterm newborn lamb model, which mimics a clinical preterm newborn infant with RDS. Survanta was purchased for a comparison surfactant because Survanta has been exclusively used in our Perinatal Institute. Commercially available surfactants and CHF5633 do not contain the collectin family members SP-A and SP-D, which play important roles in host defense [7]. On the other hand, both SP-B [8], [9], [10], and POPG [11], [12] inhibit lung inflammation and CHF5633 contains both SP-B analogue and a high amount of POPG. Therefore, the lung inflammation and ultrastructure of exogenous surfactant were studied in the CHF5633 and Survanta treated preterm newborn lamb, in addition to the physiological lung function.

**Figure 1:**
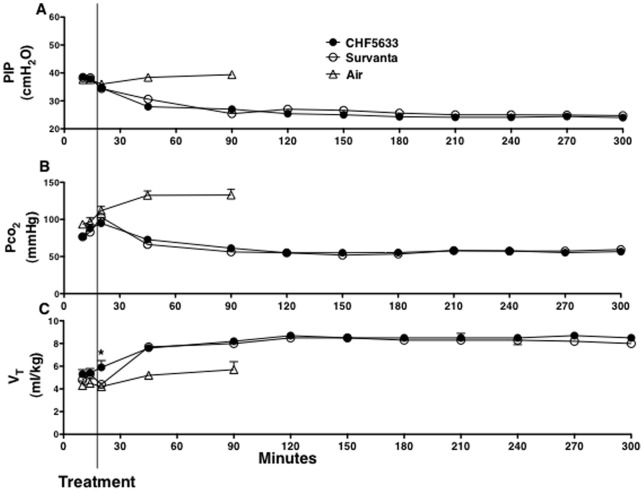
Peak inspiratory pressure (PIP), tidal volume (V_T_), and pCO_2_. Preterm newborn lambs were resuscitated after birth with (A) high peak inspiratory pressure (PIP) to regulate (C) tidal volume (V_T_) at 6 ml/kg. CHF5633, Survanta, or Air was given at 15 min of age and ventilation was changed to regulate (B) Pco_2_ at 50–60 mmHg. Lung immaturity was comparable between the groups and all the control (Air) lambs died within 90 min of age. PIP and Pco_2_ were similarly improved by treatment with CHF5633 and Survanta. Initial response of V_T_ to surfactant treatment was faster by CHF5633 than Survanta. *p<0.03. Some error bars are within symbols.

**Figure 2:**
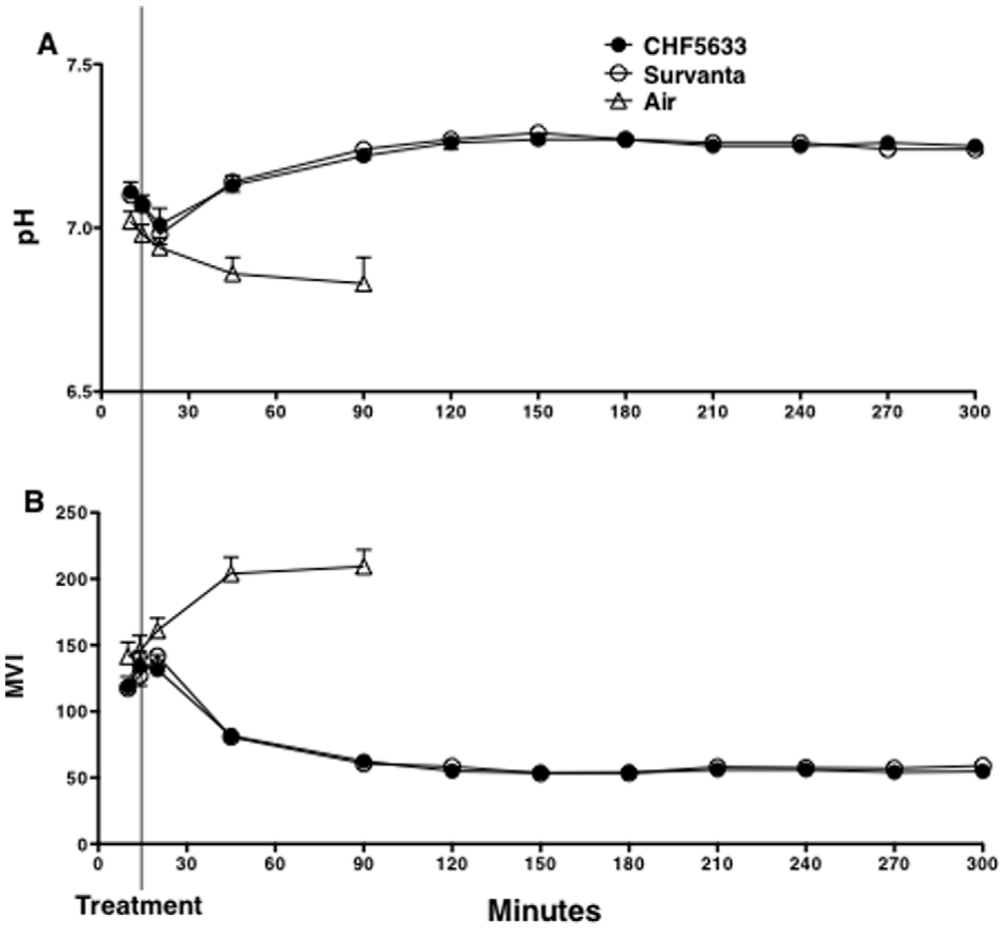
pH and modified ventilation index (MVI). (A) In spite of ventilation with high PIP for 15 min after birth, pH was decreased in association with high Pco_2_. pH was improved after CHF5633 or Survanta treatment. (B) MVI was calculated as peak inspiratory pressure x Pco_2_ x respiratory rate/1,000. MVI was similarly improved by CHF5633 and Survanta treatment. Some error bars are within symbols.

## Results

### Preterm Lamb Delivery and Ventilation

Lambs delivered at 124d gestational age (term 150d) by Cesarean section were chosen for this study because they were relatively uniform in severity of lung immaturity with surfactant deficiency. Lambs were randomly divided into 3 study groups by treatment: CHF5633, Survanta, and Air, and were treated at 15 min of age. For both surfactants, treatment doses recommended by the company were used for this translational study and were: CHF5633 200 mg/kg (2.5 ml/kg body weight, 80 mg/ml) and Survanta 100 mg/kg, (4 ml/kg body weight, 25 mg/ml). Seven lambs were studied for the CHF5633 and Survanta groups, and 5 lambs were studied for the Air group. Body weight (2.8±0.1 kg), and cord blood pH (7.40±0.03) were similar between groups. Lambs treated with air died of severe respiratory distress within 90 min of age. Therefore, the Air group was not included in the statistical analyses. Blood pressure, heart rate, hematocrit, sodium, potassium, and calcium in the blood samples were recorded every 30 min and were normal for both CHF5633 and Survanta groups throughout the study period (data not shown). Ventilation was started with the following settings: fraction of inspired oxygen (FiO_2_) = 1.0, respiratory rate = 40 breaths/min, inspiratory time = 0.7 s, positive end-expiratory pressure (PEEP) = 4 cmH_2_O, and peak inspiratory pressure (PIP) sufficient to yield 6 ml/kg tidal volume (V_T_), but with PIP limited to 40 cmH_2_O.

**Figure 3:**
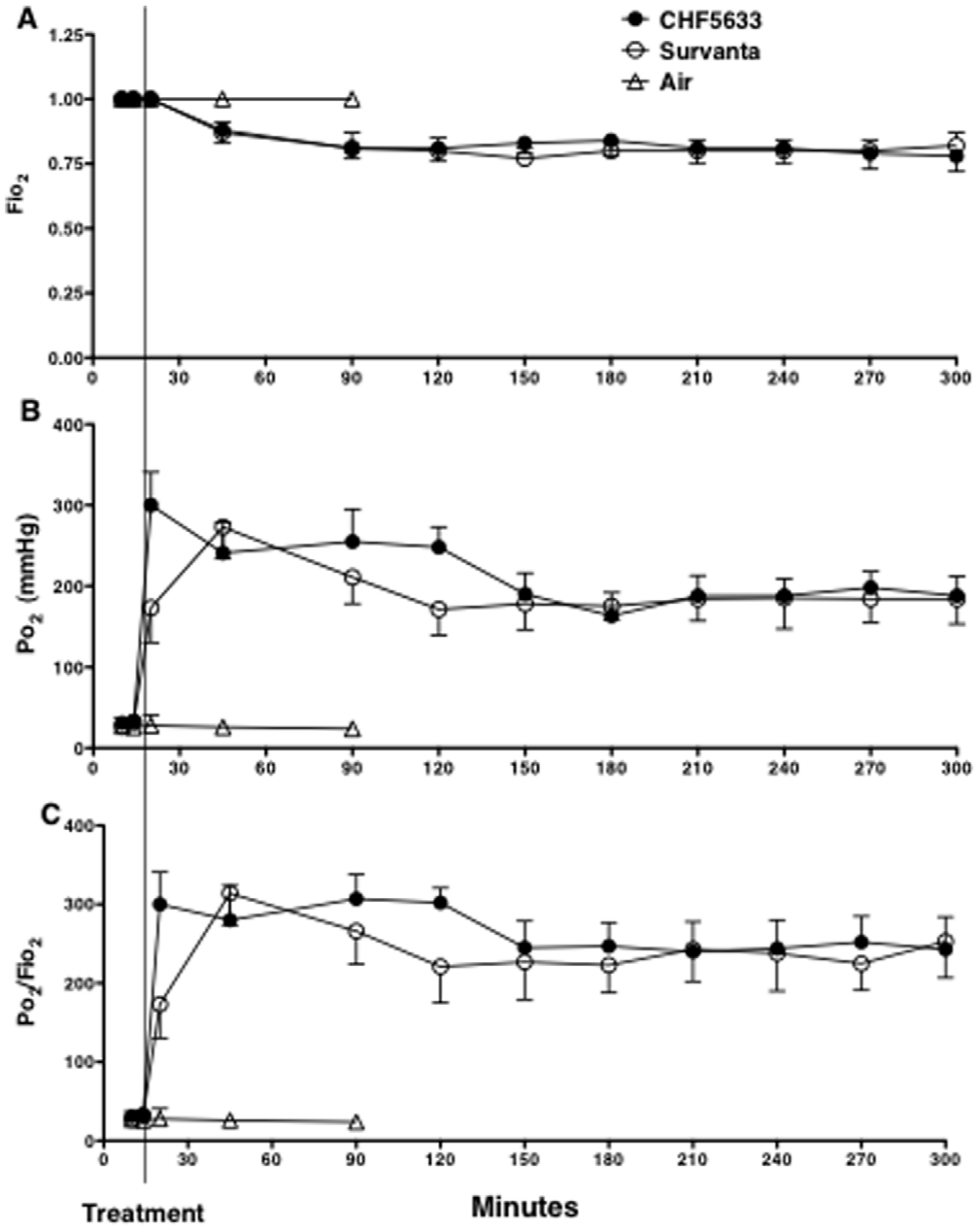
Oxygenation. These preterm newborn lambs required high Fio_2_ (A) to maintain Po_2_ (B) at targeted level. Ratio of Po_2_ to Fio_2_ (C) was improved soon after surfactant treatment. Although not significant, the increase in Po_2_/Fio_2_ after treatment tends to be faster by CHF5633 than Survanta (p = 0.052 at 20 min).

**Figure 4:**
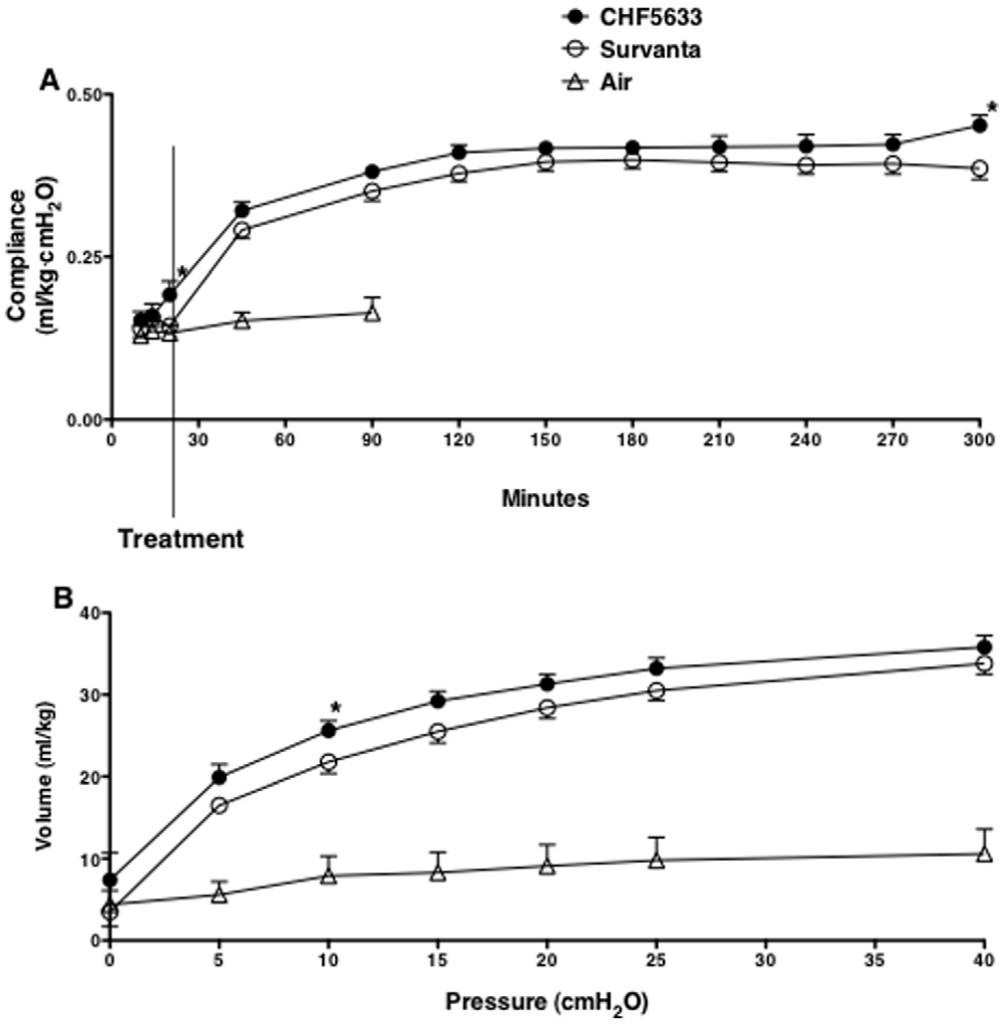
Lung compliance. (A) Lung compliance of the preterm newborn lamb was improved by both CHF5633 and Survanta treatment. Overall compliance after treatment was not significantly different between CHF5633 and Survanta group (p>0.05 by two-way repeated measures ANOVA). Compliance was higher for CHF5633 than Survanta group at 20 min and 300 min of age (*p<0.04 by t-test). (B) The overall deflation limbs of pressure-volume curves were similar between CHF5633 group and Survanta group (p>0.05 by two-way repeated measures ANOVA). The volume at 10 cmH_2_O was higher for CHF5633 group than Survanta group (*p<0.05 by t-test).

### PIP, Pco_2_, V_T_, pH, and MVI

Although lambs were resuscitated with high PIP of 38 cmH_2_O for 15 min after birth (Fig. 1A), Pco_2_ was over 80 mm Hg (Fig. 1B), and V_T_ was only 5.3 ml/kg (Fig. 1C), suggesting that these lamb lungs were extremely immature. Immediately before surfactant treatment, PIP was reduced to 35 cm H_2_O to avoid pneumothorax (Fig. 1A). The improvement of V_T_ by CHF5633 treatment was somewhat faster and V_T_ (Fig. 1C) at 20 min of age or 5 min after surfactant treatment was significantly higher than Survanta (p<0.03), suggesting faster distribution of CHF5633. After surfactant treatment, ventilation was well regulated according to the protocol. PIP and FiO_2_ were adjusted to achieve an arterial carbon dioxide tension (Pco_2_) of 50 to 60 mmHg and an arterial oxygen tension (Po_2_) of 150 to 200 mmHg with a V_T_ of 7 to 9 ml/kg. These extremely immature lambs were maintained at relatively high PO_2_ to try to close the patent ductus arteriosus. Respiratory rate, inspiratory time, and PEEP were not changed. Pco_2_ was decreased to 50–60 mmHg by 1h after surfactant treatment (Fig. 1B) and was associated with pH improvements for both groups (Fig. 2A). Modified ventilation index (MVI, PIP x Pco_2_ x respiratory rate/1,000), was likewise similarly decreased for both groups (Fig. 2B).

**Figure 5:**
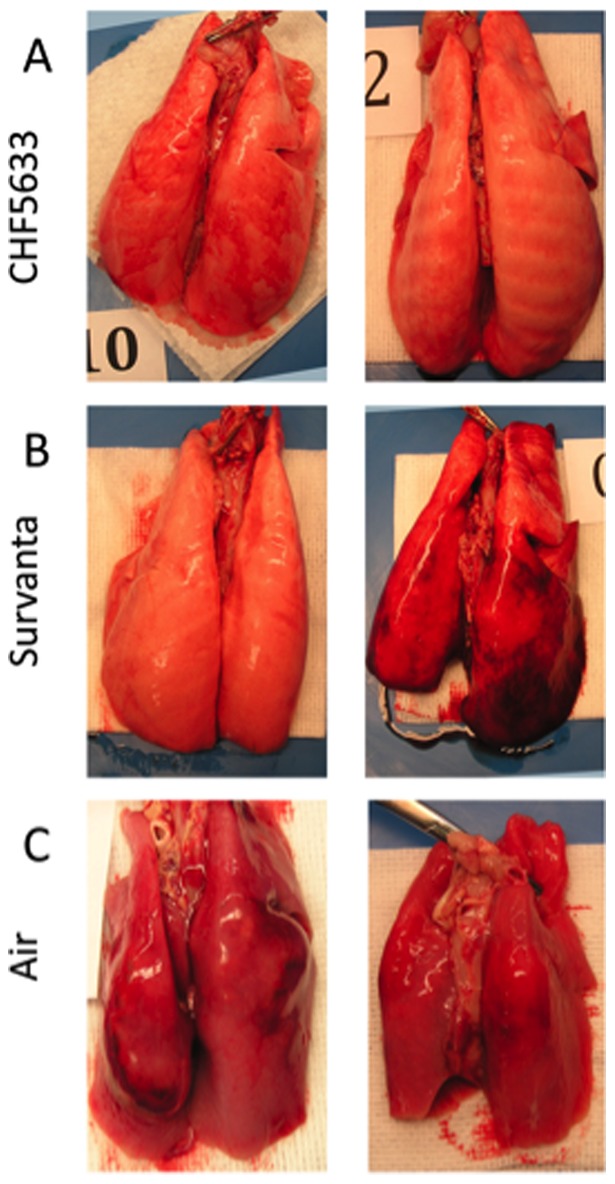
Lung appearance. Representative appearance of the lung for CHF5633 group (A), Survanta group (B), and Air group (C). CHF5633 expanded lungs uniformly for the most part. Half of the Survanta treated lambs had dark colored injured and small hemorrhagic areas in the lung. The lungs of the Air group were atelectatic with bullas.

**Figure 6:**
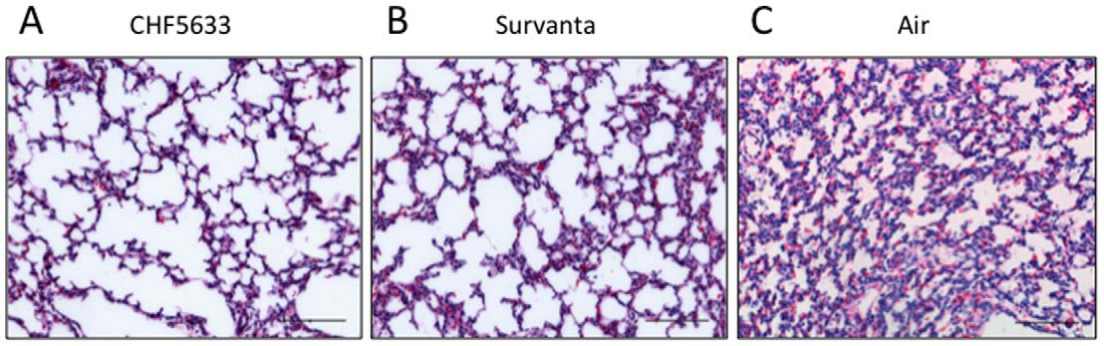
Lung Histology. Lung histology of peripheral area assessed after staining with hematoxylin and eosin. Both CHF5633 (A) and Survanta (B) groups showed various size alveoli with patchy atelectasis. The lung of the Air group (C) was atelectatic. Scale bar: 100 µm.

### Oxygenation, Lung Compliance, and Pressure-Volume Curve

High Fio_2_ (Fig. 3A) was required to maintain Po_2_ at the target (Fig. 3B) and Po_2_/Fio_2_ was not different between the CHF5633 and Survanta groups (Fig. 3C). Although not significant, oxygenation improvement after treatment tended to be faster by CHF5633 than Survanta (p = 0.052 at 20 min of age, or 5 min after treatment). Overall comparison of dynamic lung compliance during the 5h study period was similar between the CHF5633 and Survanta groups (p = 0.1 by two-way repeated measures ANOVA) (Fig. 4A). When the two groups were compared for each time point by t-test, lung compliance of the CHF5633 group was higher at 20 min and 300 min of age than Survanta group (p<0.04). On pressure-volume curve, lung volume at 10 cmH_2_O was higher for the CHF5633 group than for the Survanta group (p<0.05 by t-test), while overall comparison of the pressure-volume curve was not significantly different between the 2 groups (p = 0.07 by two-way repeated measures ANOVA) (Fig. 4B). Taken together, CHF5633 tended to improve lung compliance slightly better than Survanta.

**Figure 7:**
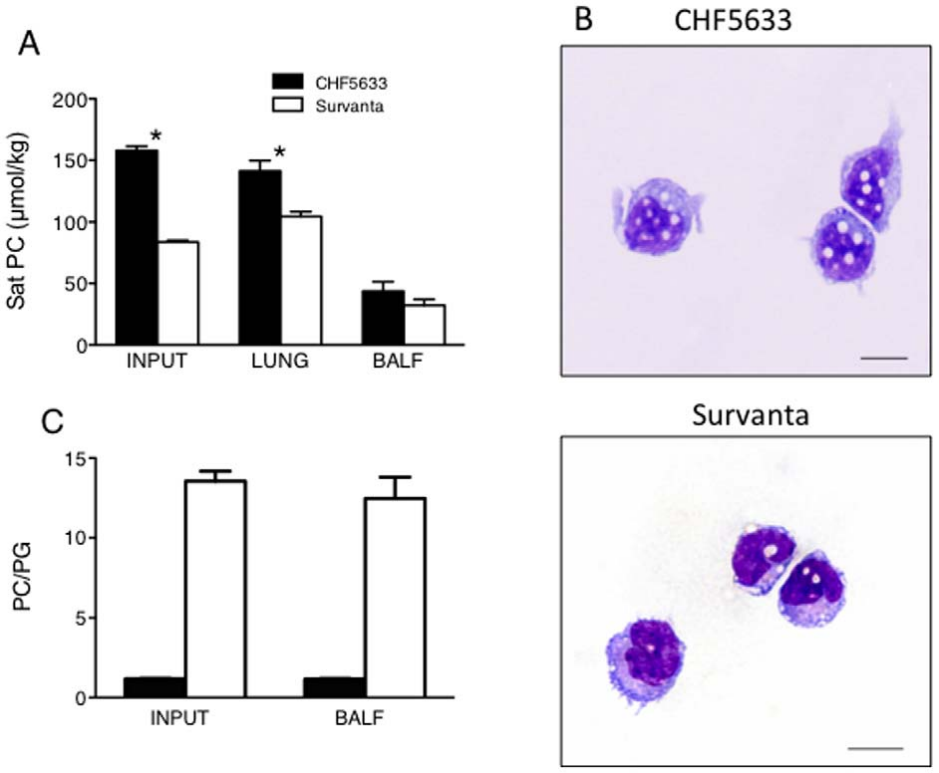
Surfactant lipid component and its uptake by alveolar monocytes. (A) Saturated phosphatidylcholine (Sat PC µmol/kg) in CHF5633 (Input), Survanta (Input), lamb lung, and BALF were analyzed. Two fold higher Sat PC was instilled to CHF5633 group than Survanta group and Sat PC in the lung was higher for CHF5633 group than Survanta group. *p<0.001 vs. Survanta In contrast, Sat PC in BALF was similar between the 2 groups, suggesting increased CHF5633 catabolism compared with Survanta. (B) Microphotograph of alveolar cells. Phagocytosis of surfactant by monocytes was higher for the CHF5633 group and monocytes contained lipid droplets. Scale bar: 10 µm. (C) Phosphatidylcholine (PC) and phosphatidylglycerol (PG) were analyzed by thin layer chromatography. There were similar amounts of PC and PG in CHF5633 and the major component of Survanta was PC. The ratio of PC to PG did not change in the ventilated preterm lamb lung and was similar to Input samples.

### Lung Appearance and Morphology

The photograph of the lung appearance demonstrated relatively uniform lung expansion by treatment with CHF5633 (Fig. 5A). There were small, injured, hemorrhagic areas with dark color in 3 out of 7 Survanta group lamb lungs (Fig. 5B). Air group lamb lungs were atelectatic and had bullas in 4 out of 5 lambs (Fig. 5C). In the two surfactant treatment groups, the center portion of the lung received a large amount of instilled surfactant and had relatively uniform well-expanded alveoli. Therefore, lung morphology was evaluated on 2 sections taken from the peripheral portions, where alveolar size would be influenced by efficacy of exogenous surfactant distribution. There were large variations in the alveolar size with patchy atelectasis in the peripheral lung of both CHF5633 and Survanta groups (Fig. 6A, B). The Air group lung was uniformly atelectatic (Fig. 6C).

**Figure 8:**
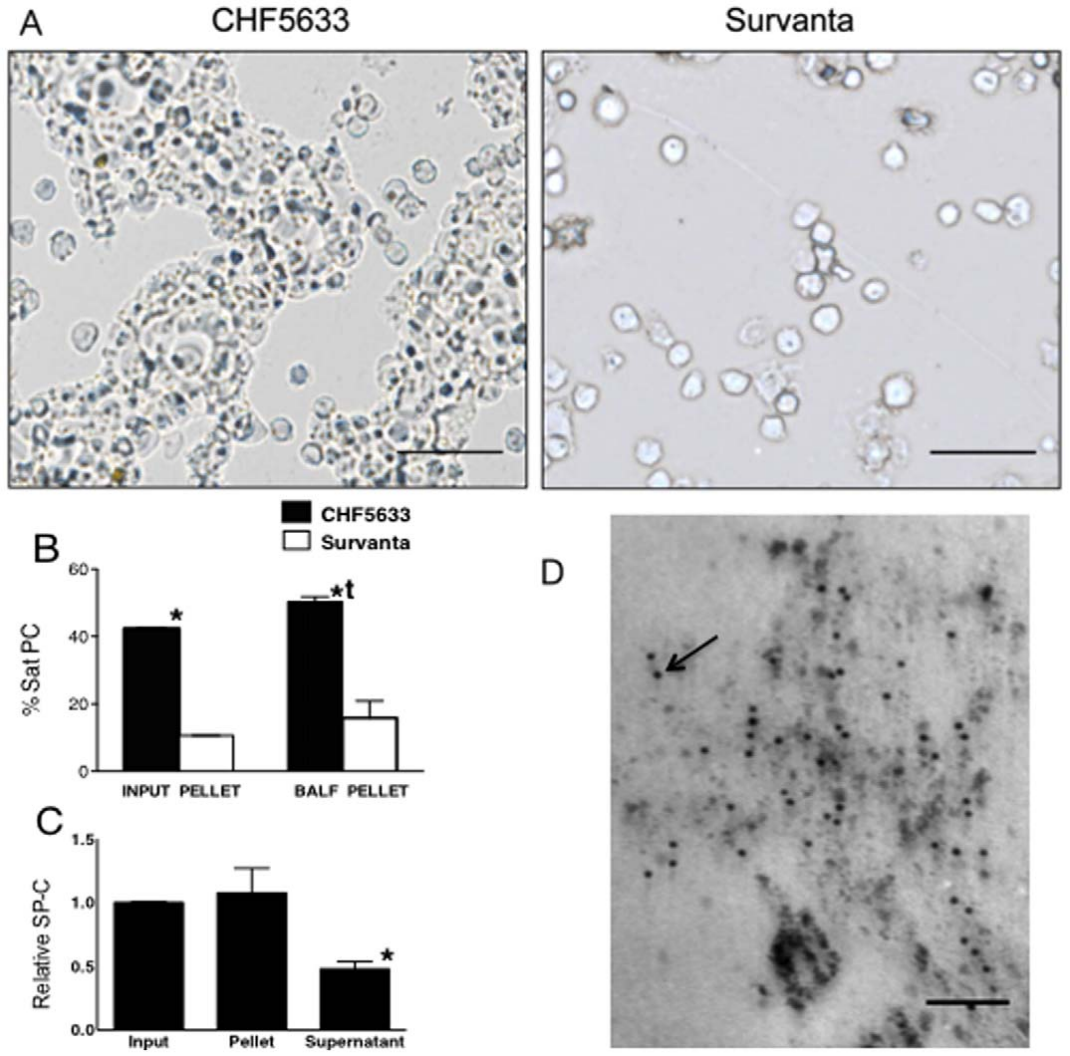
Increased Uptake of CHF5633 by Alveolar Monocyte. (A) Cell pellet from BALF isolated by 10 min centrifugation at 284× g. The microphotographs were without fixation or staining. Extremely large and soft aggregate of CHF5633 was recovered in the pellet together with cells. Detected Survanta in the cell pellet was minimum after short and low-speed centrifugation and only the cells were detected. Scale bar: 50 µm. (B) Sat PC in pellet and supernatant of input surfactants and BALFs were analyzed. Over 40% of CHF5633 were large and heavy aggregates and were recovered in the pellet. *p<0.001 vs. Survanta. The percent Sat PC in the pellet from the CHF5633 group BALF was higher than that of Input CHF5633. tp<0.05 vs. input CHF5633 pellet. Some of the standard error bars are within the mean value bars. (C) SP-C in pellet and supernatant samples of BALF from CHF5633 group, containing 6.5nmol Sat PC were analyzed by Western blot. SP-C in CHF5633 input sample was given a value of 1. SP-C relative to Sat PC was decreased in supernatant or smaller form surfactant to half of that in input sample and BALF pellet samples. *p<0.05 vs. others. (D) In the surfactant pellet of BALF from the CHF5633 group, a large number of immunogold-labeled SP-C particles (arrow) were associated with surfactant lipid vesicles. Image is representative of findings in n = 3 lambs. Scale bar: 0.1 µm.

### Surfactant Lipids

The endogenous saturated phosphatidylcholine (Sat PC) pool size of 124d GA lamb lung was analyzed in the Air group and was 40.7±2.4 µmol/kg, in which secreted Sat PC in the BALF was only 0.16±0.05 µmol/kg. The Sat PC (µmol/kg body weight) in the treatment dose of CHF5633 was 2-fold higher than Survanta (Fig. 7A, Input) and Sat PC in the lung was likewise higher for the CHF5633 group than that of the Survanta group (Fig. 7A, Lung). In contrast, Sat PC in BALF of the CHF5633 and Survanta groups were similar (Fig. 7A, BALF), suggesting increased clearance of the CHF5633 from the airways. Both alveolar type II cells and alveolar macrophages phagocytize and then catabolize (by type II cell and macrophage) or recycle (by type II cell) surfactant. There were no mature macrophages in the 124d GA newborn lamb BALF, however there were monocytes with large nuclei relative to the cell size. Alveolar type II cells are immature in the preterm newborn lung and result in the slower clearance of surfactant compared to the mature lung [13]. Over 60% of instilled surfactant remained in the lung for both groups 4.75 h after treatment (CHF5633: 64±5%, Survanta 76±5%, p>0.05). Interestingly, the alveolar monocytes in the CHF5633 group contained higher amounts of lipid droplets (Fig. 7B, white dot). Phagocytosis of CHF5633 by monocyte was increased over Survanta, which may be caused by the higher treatment dose of CHF5633. The ratio of PC to PG in input and BALF samples was analyzed. The content of PG was similarly high as PC in CHF5633 and PC/PG was 1/1, while PG was lower in Survanta (Fig. 7C). The surfactant lipid composition of instilled surfactant was unchanged in the ventilated preterm newborn lamb lung and the PC/PG ratio in BALF was similar to that of input samples for both CHF5633 and Survanta groups.

**Figure 9:**
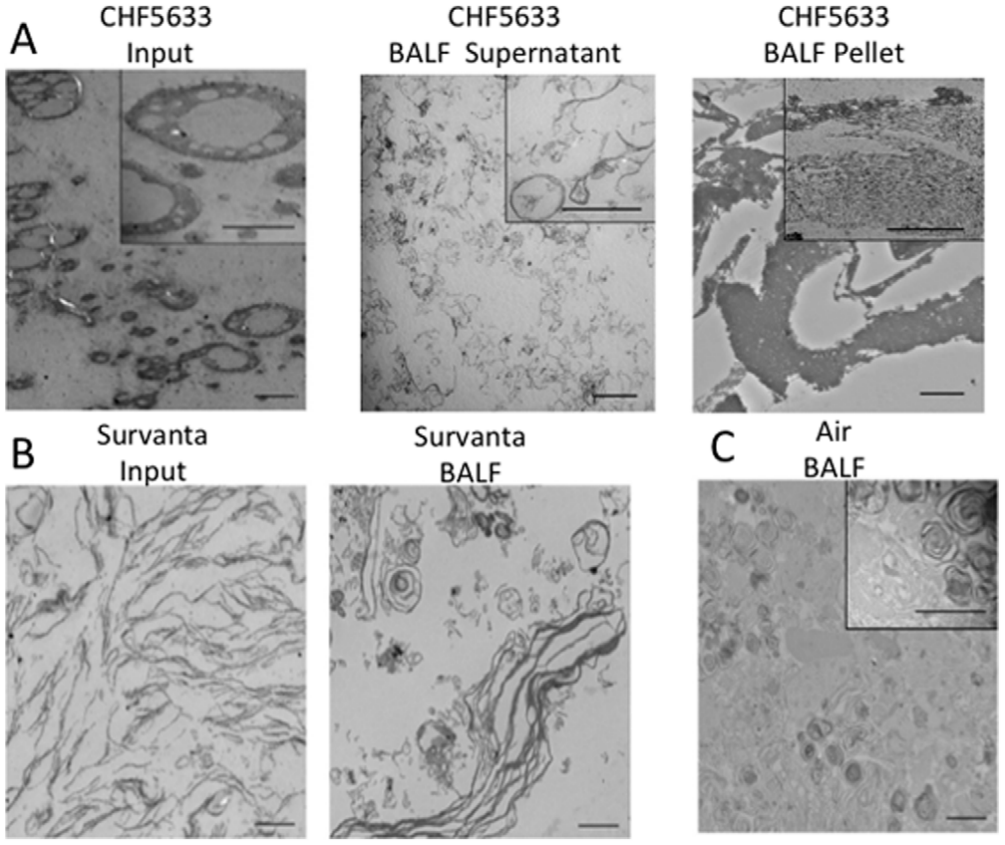
Surfactant ultrastructure. (A) CHF5633. Ultrastructure of CHF5633 Input was extremely large with ball bearing like form. In the supernatant of the BALF, ultrastructure of small aggregate of CHF5633 was lipid layers with various forms. The recovered CHF5633 in the pellet from BALF were large, softly aggregated lipid particles. (B.) Ultrastructure of Survanta was simple lipid layers that were changed to small lipid vesicle or lamellated structure in the preterm lamb lung. (C.) Air group or Endogenous Surfactant. In the extremely preterm lamb lung airways, small amounts of surfactant with normal ultrastructure including lamellar bodies and tubular myelin were present. Scale bar: 2 µm: Scale bar in inset: 0.5 µm.

### Surfactant Aggregates and SP-C in BALF

When inflammatory cells were isolated from BALF by 10 min centrifugation at 284 g, a large amount of CHF5633 was recovered in the pellet together with the cells (Fig. 8A, CHF5633). In contrast, the amount of Survanta detected in the cell pellet sample was minimal after such a short and low-speed centrifugation of BALF (Fig. 8A, Survanta). Likewise, three-fold higher percent of Sat PC was in the pellet of CHF5633 group BALF (Fig. 8B BALF pellet) compared to Survanta group (p<0.001), and was higher than CHF5633 Input pellet sample (Fig. 8B CHF5633 Input pellet vs. BALF pellet, p<0.01). Relative SP-C protein in input sample, BALF pellet, and BALF supernatant containing 6.5 nmol Sat PC were analyzed for the CHF5633 group by Western blot. Amount of SP-C relative to Sat PC in input sample and BALF pellet was similar, and was decreased in BALF supernatant containing smaller form surfactant (Fig. 8C). In the pellet of BALF recovered from the CHF5633 group lamb lung, immunogold-labeled SP-C particles were associated with surfactant lipid (Fig. 8D).

**Figure 10:**
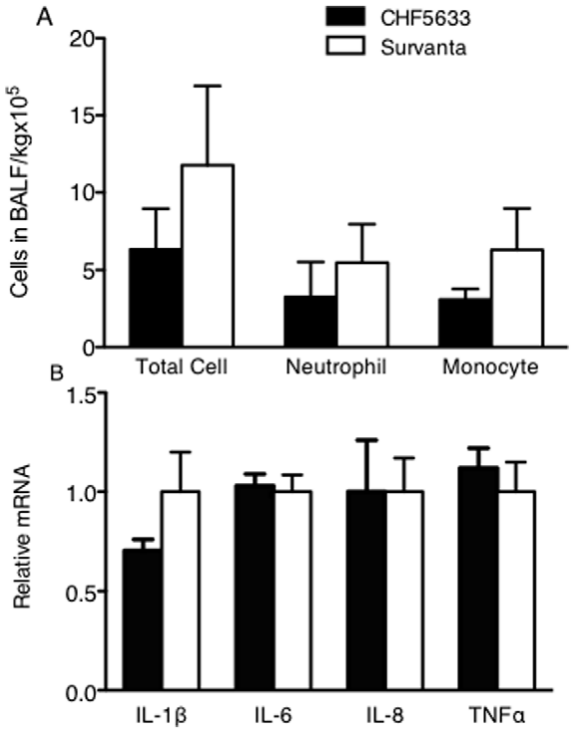
Lung inflammation. Before surfactant treatment, lambs were ventilated for 15 min with limited PIP to 40 cmH_2_O that resulted in low V_T_ of 5.3 ml/kg. Thus, extremely immature lungs with low compliance were not overstretched and lung inflammation was minimized. (A) Number of total cells, neutrophils, and monocytes in BALF were low for both groups. Although the inflammatory cells tended to be lower for CHF5633, they were not statistically different. (B) Expression of IL-1β, IL-6, IL-8, and TNFα mRNA were analyzed by RT-PCR. Although the mean value of IL-1β tended to be lower for the CHF5633 group, there were no statistical differences in IL-1β, IL-6, IL-8, and TNFα mRNA between CHF5633 and Survanta groups.

### Surfactant Ultrastructure

Surfactant for treatment changes its ultrastructure in preterm newborn lung, mainly by association with their endogenous surfactant [14]. Ultrastructure of CHF5633 was large (Fig. 9A Input) and different from that of endogenous surfactant (Fig. 9C). In the CHF5633 group, lipid layers and lipid vesicles were detected in the supernatant of BALF (Fig. 9A Supernatant) and there were large soft lumps of lipid particles in the pellet (Fig. 9A BALF Pellet). Electron microphotograph of Survanta showed simple lipid layers (Fig. 9B, Survanta Input), which changed to smaller lipid vesicles and lamellated structures in the lung (Fig. 9, BALF). Trace amount of endogenous surfactant was recovered from the Air group lamb BALF. The ultrastructure of secreted endogenous surfactant in these extremely immature lungs was normal with lamellar bodies and tubular myelin (Fig. 9C).

### Lung Inflammation

The ventilation causes lung stretch, which induces the release of inflammatory mediators and causes lung inflammation in the premature lung. Therefore, lung inflammation induced by ventilation correlates with V_T_ [5]. Due to the extremely low lung compliance of these preterm newborn lambs before surfactant treatment, lung stretch or V_T_ was low and lung inflammation induced by ventilation was minimal under our ventilation protocol. After surfactant treatment, ventilation was well managed to maintain V_T_ at the target for both groups, which resulted in similarly low lung inflammation. Total protein in BALF was low for both groups (CHF5633: 58±4 mg/kg, Survanta: 64±5 mg/kg). Number of total inflammatory cells, neutrophils, and monocytes in BALF were low (Fig. 10A) and, likewise expression of pro-inflammatory cytokine mRNAs, IL-1β, IL-6, IL-8, and TNFα, analyzed by RT-PCR were low in both CHF5633 and Survanta group lungs (Fig. 10B). In the Air group, these pro-inflammatory cytokine mRNAs were 2–7 fold higher compared to CHF5633 and Survanta groups (data not shown). Although CHF5633 contains SP-B and POPG, which are known to decrease lung inflammation [8], [9], [10], [11], [12], we could not demonstrate these functions in our present study because of the minimal lung injury in both groups.

## Discussion

Until the early 1970's, DPPC was thought to be the only critical component of surfactant [15]. Therefore, the initial clinical trials of surfactant replacement therapy for RDS were nebulization of DPPC alone and they failed [16], [17]. Later, dramatic efficacy of instilled crude surfactant on lung function improvement of prematurely delivered animal models was demonstrated [18], [19] and development of surfactant for clinical treatment was advanced. The surfactant used for the first clinical study was similar to Survanta [20] and is the modified surfactant extracted from minced bovine lung. Development of synthetic surfactant has been an interest since the 1970's [21], [22]. Up to now, 3 synthetic surfactants have been clinically tested and 14 comparison studies of animal-derived surfactant with synthetic surfactants on newborns with RDS have been published. Treatment with animal-derived surfactant preparation has been shown to result in better clinical response than synthetic surfactant during the acute phase of RDS, as evidenced by rapid weaning of inspired oxygen, lower mean airway pressure, and lower air leaks [1]. The present study demonstrated for the first time that new synthetic surfactant CHF5633 dramatically improved lung function of preterm newborn lamb lung, similarly as animal lung tissue-based surfactant. Furthermore, there were slight but significant beneficial effects of CHF5633 over Survanta on improvement of V_T_ and compliance immediately after treatment, increase in lung compliance at 300 min and higher volume of pressure volume curve at 10 cmH_2_O.

The alveolar surface is overlaid with a pulmonary surfactant film, which reduces surface tension occurring on the alveolar surface. SP-B and SP-C are hydrophobic peptides that play important roles in the formation, maintenance, and function of the surfactant surface film. Recently, the different in vivo functions between SP-B and SP-C were demonstrated using transgenic mice models. SP-B deficiency in mice and humans caused lethal respiratory dysfunction that was accompanied by failure to process pro-SP-C to its functional mature SP-C form [23], [24]. Thus, SP-B knockout mice did not clarify the distinct functions of SP-B and SP-C in vivo. Studies with a transgenic mouse model in which the human SP-B cDNA was expressed in the alveolar epithelium under conditional control of a doxycycline-inducible transgene, demonstrated that SP-B deficiency is sufficient to cause lung dysfunction in adult mice without altering SP-C content [9]. Generally speaking, SP-B is more effective in lowering minimum surface tension whereas SP-C is more effective in stabilizing the surfactant surface film [25], [26] by recruitment of surfactant lipids into the expanding surfactant film from a surfactant reservoir in the sub-phase [27], [28]. Increased improvement of lung function by the synthetic surfactant containing both SP-B and SP-C analogues, over that with a single surfactant protein, have previously been suggested [29], [30]. CHF5633 contains both SP-B and SP-C analog, whereas Survanta contains SP-C and only a trace amount of SP-B (0–1.3 mg/mM phospholipid) [1]. Palmitic acid added to Survanta play a role in surface tension lowering properties as a substitute for SP-B. There is no perfect way to compare 2 surfactant products with different components and doses. In the present study, CHF5633 and Survanta were studied using their clinical dose on the premature lamb model as the translational study.

The present study suggests that catabolism of CHF5633 may be increased in the alveolus compared to Survanta. The large amount of lipid droplet in the alveolar monocyte was demonstrated in the CHF5633 group. Likewise, the Sat PC level in BALF from both CHF5633 and Survanta groups was similar despite the 2-fold higher treatment dose of CHF5633 instilled. The ratio of PC/PG was similar between Input and BALF samples, suggesting that surfactant uptake and catabolism of CHF5633 was not selective for specific phospholipid. Alveolar macrophages increased their lipid uptake and surfactant catabolism when alveolar surfactant pool size was increased by exogenously instilled surfactant [31]. The clinical dose of CHF5633 determined by the company was 2-fold higher than Survanta, and also 2-fold higher than the surfactant pool size of normal term newborns. A newborn lung has 10-fold higher surfactant per body weight than an adult, which decreases to the normal adult level after 1wk of age [32]. Thus, both the CHF5633 and Survanta groups received sufficient amounts of surfactant in the airways and CHF5633, with a higher excess amount, may be catabolized more by monocytes than Survanta.

Surfactant ultrastructure is influenced by its components, particularly surfactant proteins [33], [34]. Survanta contains SP-C, but SP-B content in Survanta is minimal, which results in an ultrastructure of simple lipid layers. Changes in exogenous surfactant ultrastructure in the immature lung were likely caused by association with endogenous surfactant [14] and mechanical surface area changes by respiration [35]. The ultrastructure of CHF5633 was large with loosely aggregated lipid particles and different from Survanta or endogenous surfactant. Surfactant ultrastructure influences phagocytosis of surfactant by alveolar type II cells [32], [36]. SP-B caused aggregation and fusion of the liposomes to form large lipid structures and SP-C affected liposome size [36]. Although uptake of loosely aggregated surfactant particles of CHF5633 by alveolar monocyte was demonstrated in the present study, its phagocytosis by type II cells remains unknown. In the normal lung, surfactant is secreted as large aggregate that is surface active. After the formation of surfactant film over the alveolar surface, surfactant lipid is dissociated from surfactant proteins and converted to small surfactant lipid vesicles, which is the form to be catabolized. Content of SP-C protein relative to Sat PC was similar between input CHF5633 and BALF pellet samples and was decreased in BALF supernatant containing smaller surfactant aggregate. Similar to normal endogenous surfactant form conversion and surfactant protein dissociation after secretion, changes in form and surfactant protein content occurred for CHF5633 in the alveolus.

Animal-driven surfactant is made from extracted lipid of BALF or lung tissue and lipid extraction procedure remove most of the microorganism and endotoxin. Since animal-driven surfactant became available for treatment, there have been no reports of infection caused by surfactant treatment. The multiple benefits of synthetic surfactant are widely recognized, therefore abundant supply and possible lower cost might be highly rewarding. Inhibition of lung inflammation by PG was recently demonstrated [11], [12]. High content of PG in CHF5633 might be beneficial for prevention of chronic lung injury. Due to our study protocol on ventilation style, V_T_ was kept low before (V_T_ = 5 ml/kg) and after (V_T_ = 8 ml/kg) surfactant treatments. Therefore, lung inflammation was not caused in both groups and efficacy of CHF5633 to lung inflammation could not be demonstrated. Development of synthetic surfactant would extend the use of surfactant to other lung diseases including early stages of acute lung injury caused by infection, and could be used to enhance spreading of treatment substances in the airways by mixing with surfactant [37], which would improve efficacy of treatment.

## Methods

The study was approved by the Animal Care and Use Committee of the Cincinnati Children's Research Foundation.

### Delivery and Resuscitation

The preterm lambs at 124d gestation age (GA, term 150d) were delivered by C-section as previously described [38], [39], [40]. Each pregnant ewe was pre-anesthetized with a mixture of ketamine (20 mg/kg) and xylazine (3 mg/kg) intramuscularly, and then given spinal-epidural anesthesia (10 ml of 2% lidocaine and 0.5% bupivacaine, 1∶1, vol/vol). After exposure of the head, the preterm lamb was intramuscularly given ketamine (10 mg/kg) and acepromazine (0.1 mg/kg). A tracheal tube of 4.5 mm I.D. was inserted and tied into the trachea after local anesthesia of the anterior neck with 2% lidocaine. Fetal lung fluid was removed from the airways with a syringe as much as possible. After the umbilical cord was cut, the newborn lamb was delivered, the body was quickly dried with a towel and weighed. Ventilation was started using pressure-limited ventilators (Sechrist Industries, Anaheim, CA). V_T_ was monitored continuously (CP-100; Bicore Monitoring Systems, Anaheim, CA). The initial 15 min ventilation was designed to mimic the clinical situation of preterm newborn resuscitation in the delivery room. A size 5 Fr catheter was advanced into the aorta via an umbilical artery, and a 10 ml/kg transfusion of filtered fetal blood collected from the placenta was administered within 5 min of delivery to correct low hematocrit associated with immaturity. The ewe was killed with an overdose of pentobarbital (IV) immediately after delivery.

### Surfactant treatment and ventilation

CHF5633, Survanta, and Air were prepared and instilled at 15 min of age by one investigator and the study was performed blindly. The 5Fr feeding catheter was cut at 0.5 cm longer than the tracheal tube and surfactant or air was quickly instilled through this catheter in two equal aliquots to the lamb in the left lateral and right lateral positions respectively, followed by 30 s ventilation with reduced PIP to 35 cmH_2_O at each position [41]. Lambs were kept in the prone position for the rest of the study period. Dynamic compliance was calculated as V_T_ normalized to body weight and divided by the ventilatory pressure (PIP – PEEP). The arterial catheter was used for blood gas analysis, pH measurement, hematocrit, electrolytes (sodium, potassium, and calcium), blood pressure recording, and to infuse 10% dextrose (4 ml/kg/hr). Rectal temperature was monitored and kept at 38° to 39°C (normal temperature for sheep), with plastic body-covering wrap, heating pads and radiant heat. Supplemental ketamine (10 mg/kg) and acepromazine (0.1 mg/kg) were administered intramuscularly to suppress spontaneous breathing when necessary.

After 5 h, each animal was deeply anesthetized with 25 mg/kg pentobarbital given intravenously and was ventilated for 3 min with 100% oxygen. The endotracheal tube was clamped for 3 min to permit oxygen absorption for making the lung airless to prepare for the pressure-volume curve measurement. The animal was exsanguinated by cutting the abdominal aorta.

### Pressure-Volume Curve and Lung Processing

The thorax of the lamb was opened, the lungs were inflated with 40 cmH_2_O pressure for 30 sec and maximal volume was recorded [40]. The pressure was sequentially lowered to 25, 20, 15, 10, 5 and 0 cmH_2_O, and lung volumes were recorded. Volumes were corrected for the compliance of the apparatus. After lungs were removed from the thorax, the appearance of the lung inflated at 10 cmH_2_O was photographed. Pieces of the right lower lobe were immediately frozen in liquid nitrogen for RNA isolation, and the expression of pro-inflammatory cytokine mRNAs including IL-1β, IL-6, IL-8, and TNFα were analyzed by RT-PCR (7). Bronchoalveolar lavage (BAL) was done on the left lung by filling the lung with 0.9% NaCl at 4°C until visually distended, and was repeated five times. The BAL fluids (BALF) were pooled and aliquots were saved for measurement of total protein [42]. Erythrocytes in cell pellet isolated from BALF by 10 min centrifugation at 284× g were lysed using FCS lysing buffer (Invitrogen, Carlsbad, CA). The CD45 antibody recognizes the leukocyte common antigen present on cells of hematopoietic origin, except for erythroid cells and platelets. CD45-positive cells were isolated from BALF pellet using magnetic cell separation (Miltenyi Biotech, Auburn, CA) and total inflammatory cell numbers were counted. Furthermore, inflammatory cells were stained with Diff-Quik and cell differentiation was evaluated on 400 cells/sample.

### Surfactant Lipids and SP-C

Sat PC was isolated from an aliquot of CHF5633 (INPUT), Survanta(INPUT), BALF recovered from the left lung, lung homogenates of the right middle lobe [43], and BALF pellet after 10 min centrifugation at 284× g, using osmium tetroxide and neutral alumina [44] followed by phosphorus measurement [45]. Phospholipid contents of CHF5633 were phosphatidylcholine (PC) and phosphatidylglycerol (PG). To study the changes in phospholipid composition of surfactant in the premature lamb lung, PC and PG were isolated from BALF by thin layer chromatography (Silica Gel G, Analtech, Newark, DE). The PC and PG spot identified by iodine vapor were scraped from the plate [46] and their phosphorus contents were measured [45], followed by calculation of PC to PG ratio. SP-C protein in the samples containing 6.5 nmol Sat PC was analyzed by Western blot using a polyclonal antibody against human SP-C (Seven Hills Bioreagents, Cincinnati, OH).

### Lung Morphology, Surfactant Ultrastructure, and Immunogold labeling for SP-C

The right upper lobe was inflation-fixed at 30 cmH_2_O for morphology with hematoxylin and eosin stain. To study the ultrastructure of surfactant, aliquots of BALF were centrifuged at 284x g for 10 min to remove cells from BALF. Karnovsky fixative was added to the aliquots of BALF supernatant, cell pellet of BALF (for CHF5633 group only), CHF5633, and Survanta and kept at 3°C overnight [38]. The cell pellet from BALF of Survanta and Air groups contained only a trace amount of surfactant and its ultrastructure was not studied. Fixed surfactant samples were recovered by ultracentrifugation at 125,000× g for 4.5 h and prepared for electron microscopic studies [32]. SP-C on the ultrathin section of fixed BALF pellet samples from the CHF5633 group was labeled with 10-nm immunogold (Nanoprobes, Yaphank, NY) [47], using a polyclonal antibody against human SP-C (1∶1,000).

### Data Analysis

Each variable is expressed as a mean ± SE. Two-tailed unpaired t-tests, or two-way repeated measures ANOVA were used for making comparisons between CHF5633 and Survanta. All the data was statistically analyzed but p values were given only where significant differences (p<0.05) were found.
